# Damage control surgery for the treatment of perforated acute colonic diverticulitis: A systematic review

**DOI:** 10.1097/MD.0000000000023323

**Published:** 2020-11-25

**Authors:** Maurizio Zizzo, Carolina Castro Ruiz, Magda Zanelli, Maria Chiara Bassi, Francesca Sanguedolce, Stefano Ascani, Valerio Annessi

**Affiliations:** a Surgical Oncology Unit, Azienda Unità Sanitaria Locale-IRCCS di Reggio Emilia, Arcispedale Santa Maria Nuova di Reggio Emilia, Reggio Emilia; b Clinical and Experimental Medicine PhD Program, University of Modena and Reggio Emilia, Modena; c Pathology Unit, Azienda Unità Sanitaria Locale-IRCCS di Reggio Emilia, Arcispedale Santa Maria Nuova di Reggio Emilia, Reggio Emilia; d Medical Library, Azienda Unità Sanitaria Locale-IRCCS di Reggio Emilia, Arcispedale Santa Maria Nuova di Reggio Emilia, Reggio Emilia; e Pathology Unit, Azienda Ospedaliero-Universitaria - Ospedali Riuniti di Foggia; f Pathology Unit, Azienda Ospedaliera Santa Maria di Terni, Terni, Italy.

**Keywords:** damage control, diverticular disease, diverticulitis, open abdomen, surgery

## Abstract

**Background:**

Acute colonic diverticulitis (ACD) complications arise in approximately 8% to 35% patients and the most common ones are represented by phlegmon or abscess, followed by perforation, peritonitis, obstruction, and fistula. In accordance with current guidelines, patients affected by generalized peritonitis should undergo emergency surgery. However, decisions on whether and when to operate ACD patients remain a substantially debated topic while algorithm for the best treatment has not yet been determined. Damage control surgery (DCS) represents a well-established method in treating critically ill patients with traumatic abdomen injuries. At present, such surgical approach is also finding application in non-traumatic emergencies such as perforated ACD. Thanks to a thorough systematic review of the literature, we aimed at achieving deeper knowledge of both indications and short- and long-term outcomes related to DCS in perforated ACD.

**Methods:**

We performed a systematic literature review following the Preferred Reporting Items for Systematic Reviews and Meta-Analyzes (PRISMA) guidelines. Pubmed/MEDLINE, Embase, Scopus, Cochrane Library, and Web of Science databases were used to search all related literature.

**Results:**

The 8 included articles covered an approximately 13 years study period (2006–2018), with a total 359 patient population. At presentation, most patients showed III and IV American Society of Anesthesiologists (ASA) score (81.6%) while having Hinchey III perforated ACD (69.9%). Most patients received a limited resection plus vacuum-assisted closure at first-look while about half entire population underwent primary resection anastomosis (PRA) at a second-look. Overall morbidity rate, 30-day mortality rate and overall mortality rate at follow-up were between 23% and 74%, 0% and 20%, 7% and 33%, respectively. Patients had a 100% definitive abdominal wall closure rate and a definitive stoma rate at follow-up ranging between 0% and 33%.

**Conclusion:**

DCS application to ACD patients seems to offer good outcomes with a lower percentage of patients with definitive ostomy, if compared to Hartmann's procedure. However, correct definition of DCS eligible patients is paramount in avoiding overtreatment. In accordance to 2016 WSES (World Society of Emergency Surgery) Guidelines, DCS remains an effective surgical strategy in critically ill patients affected by sepsis/septic shock and hemodynamical unstability.

## Abstract 2

Supplemental Digital Content is available in the text

## 1 Introduction

Acute colonic diverticulitis (ACD) is defined as an acute inflammation of one or more colonic diverticula.^[1,2]^ Approximately 10% to 25% patients affected by colonic diverticulosis are going to develop ACD in their lifetime.^[1–3]^ ACD complications arise in approximately 8% to 35% patients and the most common ones are represented by phlegmon or abscess (about 70% complications), followed by perforation, peritonitis, obstruction, and fistula.^[1,4,5]^ Peridiverticular and pericolic infections stem from a microscopic or macroscopic perforation of one or more inflamed diverticula.^[1,5]^

In accordance with current guidelines, patients affected by generalized peritonitis should undergo emergency surgery.^[2]^ However, decisions on whether and when to operate ACD patients remain a substantially debated topic while algorithm for the best treatment has not yet been determined.^[1,2]^ To date, no single treatment strategy has turned out as best method, in terms of efficacy and safety.^[1,2]^

Krukowski et al and Vermeulen et al suggested a classification of surgical procedures to be performed in perforated ACD (Table 1), while neither the most recent laparoscopic lavage nor the more recent and less widespread damage control surgery (DCS) were mentioned.^[2]^

**Table 1:** Operative procedures^[2]^.

Conservative: perforated colon retained in peritoneal cavity
1. Suture of perforation
2. Drainage
3. Transverse colostomy
4. Caecostomy
5. Any combination of 1–4
Radical: perforated colon eliminated from peritoneal cavity
1. No resection
• Exteriorization
2. Resection
a. Without anastomosis
• Hartmann's procedure
• Sigmoid resection with mucous fistula
• Paul-Mickulicz procedure
b. With anastomosis
• Without defunctioning stoma
• With defunctioning stoma

DCS represents a well-established method in treating critically ill patients with traumatic abdomen injuries.^[6]^ DCS strategy includes abbreviated source-control laparotomy followed by intensive care unit (ICU) transfer for physiology resuscitation and delayed surgery for definitive management.^[6]^ At present, such surgical approach is also finding application in non-traumatic emergencies such as perforated ACD.^[7]^

Thanks to a thorough systematic review of the literature, we aimed at achieving deeper knowledge of both indications and short- and long-term outcomes related to DCS in perforated ACD.

## 2 Methods

The protocol for this systematic review was registered on PROSPERO (CRD42020186958) and is available in full on the NIHR HTA programme website (https://www.crd.york.ac.uk/prospero/display_record.php?RecordID=186958).

### 2.1 Search strategy

We carried out a systematic literature review, according to Preferred Reporting Items for Systematic Reviews and Meta-Analyzes (PRISMA) guidelines.^[8]^ According to the *gold standard* for literature search for surgical reviews,^[9]^ PubMed/MEDLINE, Embase, Scopus, Cochrane Library (Cochrane Database of Systematic Reviews, Cochrane Central Register of Controlled Trials-CENTRAL), and Web of Science (Science and Social Science Citation Index) databases were used to search all related literature, by combining the following non-MeSH/MeSH terms:

- PubMed/MEDLINE
  (“Laparotomy”[Mesh] OR Open abdomen OR Surgery OR Laparotomy OR Surgical procedure OR Operative OR General surgery) AND (“Sepsis”[Mesh] OR “Peritonitis”[Mesh] OR “Abdomen, Acute”[Mesh] OR Septic shock OR Sepsis OR Peritonitis OR Acute abdomen) AND (“Diverticulitis”[Mesh] OR diverticulitis OR diverticular disease) AND (damage OR damage control)
- Embase
  (damage OR “damage control surgery”) AND (diverticulitis/exp OR Diverticulitis OR Diverticular disease) AND (sepsis OR “septic shock” OR peritonitis OR “acute abdomen”) AND (“open abdomen” OR surgery OR laparotomy OR operative OR surgical procedure∗)
- Scopus
  (TITLE-ABS-KEY (damage AND control) AND TITLE-ABS-KEY (diverticulitis) AND TITLE-ABS-KEY (open AND abdomen OR surgery OR laparotom∗) AND TITLE-ABS-KEY (sepsis OR “septic AND shock” OR peritonitis OR “acute AND abdomen”))
- Cochrane Library
  (diverticulitis OR diverticular disease) in Title Abstract Keyword AND (Septic shock OR Sepsis OR Peritonitis OR Acute abdomen) in Title Abstract Keyword AND (Open abdomen OR Surgery OR Laparotomy OR Surgical procedure OR Operative OR General surgery) in Title Abstract Keyword AND (damage OR damage control) in Title Abstract Keyword
- Web of Science
  TOPIC: (damage control) AND TOPIC: (diverticulitis) AND TOPIC: (open abdomen OR surgery OR laparotom∗) AND TOPIC: (acute abdomen OR septic shock OR peritonitis OR sepsis)
  Our final analysis was carried out in March 2020.

### 2.2 Inclusion criteria

Only English-written scientific papers were selected, including case reports, case series, case–control studies, cohort studies, controlled clinical trials, and randomized clinical trials. Prior systematic reviews and meta-analyses were ruled out. We considered both comparative and non-comparative studies including adult patients (over 18 years of age) treated for peritonitis by perforated ACD through DCS strategy as defined in “Damage control surgery procedures” paragraph. Given the lack of scientific studies on this topic, all articles of qualitative interest have been selected despite population size, publication status, and lack of interesting parameters in some of them. In addition, references of relevant articles (previously published reviews, systematic reviews or meta-analyses, and the articles included in the qualitative analysis) were searched through, in order to identify further cases of interest.

### 2.3 Data extraction

Two independent reviewers (MZ and MCB) selected and identified papers based on title, abstracts, keywords, and full-text. From the selected papers, they gathered following information: demographic and clinical data [author's surname and year of publication, study type, study period, population size, gender, and age, American Society of Anesthesiologists (ASA) score, Hinchey classification, inclusion criteria, clinical presentation, duration of peritonitis, Mannheim Peritonitis Index (MPI)]; intraoperative and perioperative data [DCS strategy at first- and second-look, operating time at first- and second-look, medical and surgical complications, ICU and hospital stays, overall morbidity, 30-day and follow-up mortalities]; open abdomen and stoma outcomes [negative pressure wound therapy (NPWT) duration, NPWT-related complications, wound closure at second-look and follow-up, ostomies at second-look and follow-up]. Eventually, all collected results were reviewed by a third independent reviewer (VA).

### 2.4 Quality assessment

The Newcastle-Ottawa quality assessment scale (NOS) was used to assess the quality of each study. Thresholds for converting the Newcastle-Ottawa scales to AHRQ standards (good, fair, and poor):

i) good quality: 3 or 4 stars in selection domain AND 1 or 2 stars in comparability domain AND 2 or 3 stars in outcome/exposure domain,
ii) fair quality: 2 stars in selection domain AND 1 or 2 stars in comparability domain AND 2 or 3 stars in outcome/exposure domain;
iii) poor quality: 0 or 1 star in selection domain OR 0 stars in comparability domain OR 0 or 1 stars in outcome/exposure domain.

### 2.5 Damage control surgery procedures

Damage control surgery divides into 5 steps:

i) identification critically ill patient according to injury pattern (underlying disease) and pathophysiology;
ii) abbreviated surgery, to control bleeding and contamination;
iii) parameter re-evaluation with patient on operating table;
iv) continued restoration of physiology at ICU;
v) definitive surgical repair.^[10]^

In a perforated ACD setting, initial emergency operation (first-look) was as short as possible and focused on source control, with limited resection of perforated colon segment, proximal and distal colon closure, leaving stapled colon without in situ reconstruction, peritoneal lavage, and temporary abdominal closure by use of NPWT during initial surgery.^[11–18]^ In selected cases, closure of perforation site was carried out through interrupted sutures, instead of performing colon resection.^[12]^

After patient resuscitation at ICU, elective second-look surgery was performed 24 to 48 hours later.^[11–18]^ In order to decide final surgical strategy – primary resection anastomosis (PRA), primary anastomosis with defunctioning stoma (PADS), or Hartmann's procedure (HP) – following aspects were taken into account: patient recovery from septic shock, clearance of peritonitis, comorbidities, and life expectancy.^[11–18]^ PADS turned out as elected method of reconstruction, although HP was performed in case of persistent severe peritonitis or septic shock.^[11–18]^ In selected cases after direct suturing of perforation site, sigmoid colon was left in place, in case colon had showed good healing at second-look surgery.^[12]^

For the NPWT, a VAC system was used (KCI, ABTHERA Therapy System; KCI, GranuFoam; KCI, VERAFLO Therapy; Lohmann & Rauscher, Suprasorb CNP drainage foam).^[11–18]^ Intraabdominal structures were covered with as much omentum as possible, while VAC-system intraabdominal part was placed into abdominal cavity and covered by non-adhesive fenestrated interface layer, in order to prevent intraabdominal damage.^[11–18]^ Uncovered foam was subcutaneously placed as second layer and sealed by adhesive film.^[11–18]^ After complete dressing, continuous negative pressure (KCI −125 mm Hg, Lohmann & Rauscher −80 mm Hg) was applied.^[11–18]^

NPWT was continued after intestinal reconstruction, in case clearance of peritonitis was inadequate, in case of abdominal compartment syndrome risk, or when surgeon deemed anastomosis re-evaluation as necessary.^[11–18]^

All procedures were performed using laparotomies.^[11–18]^

## 3 Results

### 3.1 Search results and study characteristics

**Figure 1:**
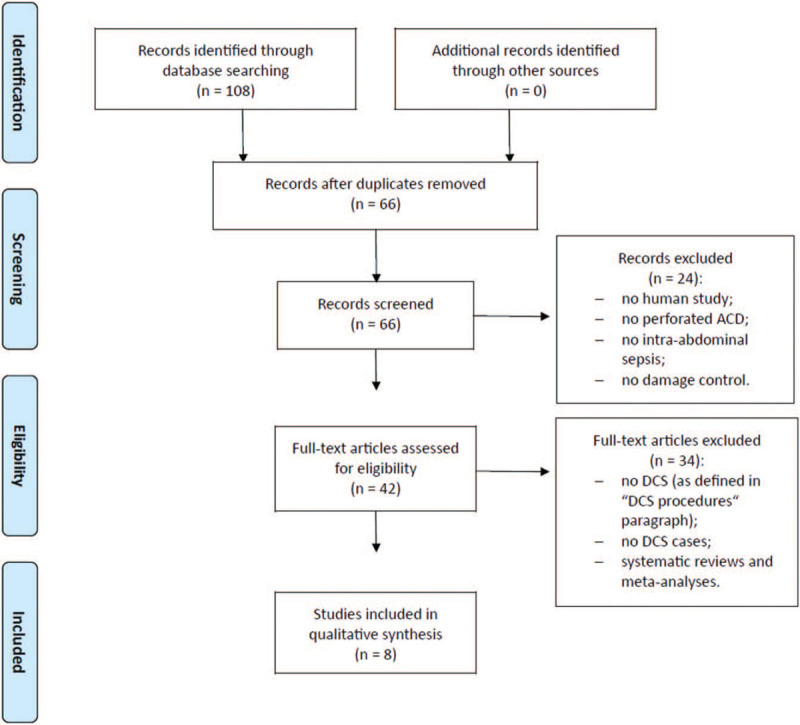
PRISMA flow chart of literature search.

Final literature search, performed in March 2020, identified 108 potential items of interest (Fig. 1). After removing duplicate publications (42), 66 records were further analyzed. Twenty-four out of which were excluded as not relevant, while 42 full-text articles were assessed for eligibility. After removing full-text articles not complying with inclusion criteria (34), 8 articles were included into qualitative synthesis.^[11–18]^ No item was included on the basis of other sources (e.g., references lists). The included articles were single-center retrospective studies (3), multicenter retrospective studies (4), and single-center prospective studies (1). Most of the studies were of good quality (see Table, Supplemental Digital Content, which illustrates the Newcastle-Ottawa Quality Assessment Form for included cohort studies).

### 3.2 General population characteristics

Table 2 shows clinical and demographic features of analyzed populations. The included 8 articles covered a 2006 to 2018 study period with a total population of 359 patients.^[11–18]^ The general population recorded a slight female prevalence (194/359 = 54%) over males and a median age between 65 and 73 years.^[11–18]^ At presentation, most patients showed III and IV ASA score (293/359 = 81.6%) while having Hinchey III perforated ACD (251/359 = 69.9%).^[11–18]^ According to available data, 7 patients had sepsis (2 out of 8 studies),^[17,18]^ 70 ones had septic shock (6 out of 8 studies),^[11,13–15,17,18]^ and 86 ones had organ failure (7 out of the 8 studies).^[11–15,17,18]^ 116 patients had peritonitis lasting longer than 24 hours (4 out of 8 studies).^[12,14,15,17]^ In addition, median MPI ranged between 16 and 26.^[11–18]^

**Table 2:** Demographic and clinical data of reported cases/series of DCS for perforated acute colonic diverticulitis.

				Gender, n (%)			ASA score, n (%)				Hinchey, n (%)				Clinical presentation, n (%)					
Author/Year	Study type	Study period	DCS patients, n	Male	Female	Age, median (range)	I–II	III	IV	V	I–II	III	IV	Inclusion criteria	Sepsis	Septic shock	Lethal triad	Organ failure	Peritonitis > 24 h, n (%)	MPI, median (range)
Perathoner et al/2010^[11]^	PS	2006–2008	15	7 (47)	8 (53)	66 (50–81)	None	11 (73)	3 (20)	1 (7)	None	12 (80)	3 (20)	Hinchey III/IV	NA	5 (33)	NA	5 (33)	NA	22 (17–35)
Kafka-Ritsch et al/2012^[12]^	RS	2006–2011	51	23 (45)	28 (55)	67 (28–69)	None	8 (16)	42 (82)	1 (2)	None	40 (78)	11 (22)	Hinchey III/IV	NA	NA	NA	16 (31)	41 (80)	26 (12–39)
Sohn et al/2016^[13]^	RS	2010–2015	19	6 (32)	13 (68)	72.6 (NA)	NA	15 (79)		NA	None	17 (89)	2 (11)	Hinchey III/IV	NA	5 (26)	NA	5 (26)	NA	16 (NA)
Sohn et al/2018^[14]^	RS	2011–2017	74	34 (46)	40 (54)	66.2 (30–92)^∗^	NA	58 (78)		NA	None	60 (81)	14 (19)	Hinchey III/IV	NA	16 (22)	NA	16 (22)	41 (55)	22.4 (6–42)^∗^
Sohn et al/2018^[15]^	RS	2011–2017	58	30 (52)	28 (48)	70 (30–92)	NA	50 (86)		NA	None	47 (81)	11 (19)	Hinchey III/IV	NA	9 (16)	NA	9 (16)	30 (52)	21.5 (6–42)
Gasser et al/2019^[16]^	RS	2009–2014	78	38 (49)	40 (51)	65 (30–90); 67 (43–86)	23 (29)	49 (63)	6 (8)	None	9 (11)	49 (63)	20 (26)	Hinchey III/IV	NA	NA	NA	NA	NA	22 (0–33); 22 (11–39)
Brillantino et al/2019^[17]^	RS	2016–2018	30	12 (40)	18 (60)	68.5 (35–84)	None	18 (60)	11 (37)	1 (3)	None	13 (43)	17 (57)	Hinchey III/IV; ASA >=3	7 (23)	1 (3)	NA	1 (3)	4 (13)	26.2 (12–40)
Tartaglia et al/2019^[18]^	RS	2011–2017	34	15 (44)	19 (56)	66.9 ± 12.7^∗^	12 (35)	22 (65)			None	13 (38)	21 (62)	Hinchey III/IV	None	34 (100)	NA	34 (100)	NA	25.12 ± 6.28^∗^

### 3.3 Damage control surgery

Table 3 shows available data about DCS strategy. Most patients received a limited resection plus NPWT at first-look (260 patients in 6 out of 8 studies).^[12–15,17,18]^ At a second-look, about half entire population underwent PRA (183/356 = 51%), while 23% patients underwent PADS and 25% patients underwent HP.^[11–18]^ Three patients died before second-look.^[11–18]^

**Table 3:** Intraoperative and perioperative outcomes data of reported cases/series of DCS for perforated acute colonic diverticulitis.

Author/Year	DCS at first-look, n (%)		Surgical strategy at second-look, n (%)					Operative time (minutes), median (range)				Surgical complications	Medical complications				
	Suture+VAC	Resection+VAC	Suture	PRA	PADS	HP	Interval between first-look and second-look (days); median (range)	At first-look	At second-look	ICU stay (days), median (range)	Hospital stay (days), median (range)	Overall (first-look+second-look)	Overall (first-look+second-look)	Patients undergoing extra-DCS reoperation for complications, n (%)	Overall morbidity, n (%)	30-day Mortality, n (%)	Overall Mortality at follow-up, n (%)
Perathoner et al/2010^[11]^	NA	NA	None	9 (60)	None	6 (40)	NA [1 or 1.5]	100 (60–210)	NA	5 (1–30)	NA	Anastomotic leakage (1); Abdominal wall dehiscence (2); Wound infection (3); Intraabdominal abscess (3)	Catheter-related infections (2); Urinary tract infections (2); Pneumonias (2); Pancreatitis (1).	2 (13)	NA	3 (20)	5 (33)
Kafka-Ritsch et al/2012^[12]^	6 (12)	45 (88)	3 (6)	31 (62)	4 (8)	12 (24)	NA [1 or 2]	85 (NA)	120 (NA)	6 (1–42)	24 (9–71)	Anastomotic leakage (5); Intraabdominal abscess (2)	NA	7 (14)	NA	5 (10)	8 (16)
Sohn et al/2016^[13]^	None	19 (100)	None	11 (58)	4 (21)	4 (21)	NA [1 or 2]	96 ± 42^∗^		2 (0–17)	18 (3–37)	Anastomotic leakage (1); Wound infection/dehiscence (4); Intraabdominal abscess (1)	NA	1 (5)	6 (32)	2 (11)	2 (11)
Sohn et al/2018^[14]^	None	74 (100)	None	37 (50)	25 (34)	12 (16)	2.1; 1.9^∗^	96 (41–210)^∗^	NA	4.6; 9.9^∗^	22 (3–66)^∗^	Anastomotic leakage (8); Abdominal wall dehiscence (5); Wound infection (12); Intraabdominal abscess (1); Intraabdominal bleeding (1)	NA	NA	26 (35)	NA	5 (7)
Sohn et al/2018^[15]^	None	58 (100)	None	34 (59)	14 (24)	10 (17)	2 (1–4)	95 ± 35.5^∗^	95 ± 35.5^∗^	NA	18.5 (3–66)	Anastomotic leakage (6); Abdominal wall dehiscence (10); Wound infection/dehiscence (10); Intraabdominal abscess (1)	NA	NA	22 (34)	0 (0)	5 (9)
Gasser et al/2019^[16]^	NA	NA	None	16 (20)	30 (40)	30 (40)	NA [1 or 2]	NA	NA	6 (0–55); 6 (2–46)	22 (1–126); 25 (8–75)	Anastomotic leakage (10); Abdominal wall dehiscence (6)	NA	NA	31 (61); 20 (74)	15 (19)	NA
Brillantino et al/2019^[17]^	None	30 (100)	None	24 (80)	None	6 (20)	NA [1 or 2]	92 (45–135)	NA	NA	18 (12–62)	Anastomotic leakage (1); Wound infection (3); Intraabdominal abscess (1)	Pneumonias (2)	1 (3)	7 (23)	1 (3)	NA
Tartaglia et al/2019^[18]^	None	34 (100)	0 (0)	21 (62)	3 (9)	10 (29)	2 (NA)	NA	NA	13.98 ± 13.47^∗^	21.9 ± 16.24^∗^	Anastomotic leakage (1); Abdominal wall dehiscence (2)	NA	3 (9)	14 (41)	4 (12)	4 (12)

Almost all cases recorded a 24 to 28 hours time lapse between first-look and second-look and a 85 to 120 minutes median operative time.^[11–18]^

### 3.4 Perioperative outcomes

Table 3 shows available data regarding perioperative outcomes. Median length of ICU stay was between 2 and 6 days, while median hospitalization length recorded between 18 and 22 days.^[11–18]^ Anastomotic leakage, intraabdominal abscess, abdominal wall dehiscence, wound infection/dehiscence, intraabdominal bleeding were the most frequently reported overall surgical complications (first-look + second-look).^[11–18]^ Overall morbidity rate was between 23% and 74% (6 out of 8 studies).^[13–18]^ Just 5 studies reported how many patients underwent reoperations due to surgical complications, whose rate was between 3% and 14%.^[11–13,17,18]^

Thirty-day mortality rate was between 0% and 20% (7 out of 8 studies).^[11–13,15–18]^ Six out of 8 studies reported overall mortality rate at follow-up, ranging between 7% and 33%.^[11–15,18]^

### 3.5 Open abdomen and ostomy outcomes

Table 4 shows available data about open abdomen and ostomy outcomes. Just Kafka-Ritsch et al and Gasser et al reported 2 to 3 days median duration of NPWT.^[12,16]^ Four out of 8 studies declared NPWT-related lack of complications.^[11,12,16,17]^ Four studies reported 57% to 100% abdominal wall closure rate at second-look^[11,12,16,17]^ and a 100% definitive abdominal wall closure rate,^[11–13,17]^ just taking into account alive patients for both rates.

**Table 4:** Open abdomen and ostomy outcomes data of reported cases/series of DCS for perforated acute colonic diverticulitis.

Author/yr	NPWT duration (d), median (range)	NPWT-related complications, n (%)	Wound closure (OA vs SCO) at second-look/patients alive, n (%)	Wound closure (OA vs SCO)/patients alive at follow-up, n (%)	Ostomy at second-look/patients alive, n (%)	Definitive ostomy/patients alive at follow-up, n (%)
Perathoner et al/2010^[11]^	NA (NA-7)	None	15/15 (100)	10/10 (100)	4/12 (33)	0/10 (0)
Kafka-Ritsch et al/2012^[12]^	3 (2–8)	None	29/51 (57)	43/43 (100)	17/46 (37)	3/43 (7)
Sohn et al/2016^[13]^	NA	NA	NA	17/17 (100)	6/17 (35)	2/17 (12)
Sohn et al/2018^[14]^	NA	NA	NA	NA	43/74 (58)^a^	17 (23)^a^
Sohn et al/2018^[15]^	NA	NA	NA	NA	29/58 (50)	9/53 (17)
Gasser et al/2019^[16]^	3 (1–12); 2 (1–6)	None	48/76 (63)	NA	27/76 (35)	NA
Brillantino et al/2019^[17]^	NA	None	29/29 (100)	NA/NA (100)	23/29 (79)	NA
Tartaglia et al/2019^[18]^	NA	NA	NA	NA	13/30 (43)	10/30 (33)

Patients who had an ostomy at second-look varied between 33% and 79%,^[11–18]^ while those who had a definitive stoma at follow-up were between 0% and 33% (6 out 8 studies).^[11–15,18]^ For those rates just living population was taken into account.

## 4 Discussion

Diverticular perforation is an extremely important occurrence in ACD natural history. Mortality following complicated ACD (abscess, perforation, or fistula) has increased, if compared to mortality in patients affected by uncomplicated ACD.^[1]^ It records the highest rate among patients with perforation or abscess.^[1]^ A UK cohort study reported a 20% 1-year mortality rate for patients with perforated ACD, against 4% controls matched by age and gender.^[1]^

After first diverticulitis acute attack, 20% to 30% patients go to surgery, being about half of them performed at emergency.^[1–3]^ Fifteen to 40% out of these cases involve people younger than 50.^[1–3]^

To date, HP is the most performed method in Hinchey III and IV patients.^[2]^ Despite being a relatively simple and ideally safe surgical procedure and given absence of intestinal anastomosis, its morbidity and mortality are not negligible. Keep in mind that Hartmann's reversal is typified by a 49% to 55% morbidity and 20% mortality rates.^[2]^ In addition, a large amount of patients will never undergo stoma reversal (48–74%), although patients affected by diverticular disease show high stoma reversal rates (83%).^[2]^

LADIES, a multicenter, parallel, randomized, open-label superiority trial identified a 12-month stoma-free rate of 94.6% and 71.4% (Hinchey III: PADS 95.3% vs HP 79.8%; Hinchey IV: PADS 92.2% vs HP 51.9%) with a median interval of reversal of 101 days and 186 days for PADS and HP, respectively.^[4]^ In intention-to-treat analysis, no statistically significant discrepancy was identified between HP and PADS, as concerned perioperative mortality (3% vs 6%) and overall morbidity (HP 44% vs PADS 39% – Hinchey III: HP 37% vs PADS 37%; Hinchey IV: HP 60% vs 44%).^[4]^ In stoma reversal analysis, 68% HP patients and 83% PADS patients underwent stoma reversal with a median interval of reversal of 133 days and 113.5 days, for their respective groups.^[4]^ Overall morbidity recorded a statistically significant discrepancy between HP patients and PADS ones (30% vs 8%).^[4]^

DIVERTI, a multicenter, prospective, randomized controlled trial reported no statistically significant difference between HP and PADS, in terms of mortality and overall morbidity (42.3% vs 54%) at emergency surgery analysis.^[19]^ In stoma reversal analysis, discrepancy among HP patients and PADS ones turned out statistically significant (64.6% vs 96%), while overall morbidity recorded no statistically significant difference (21.2% vs 12.5%).^[19]^

Above mentioned findings are in accordance with those gathered by recent meta-analyzes.^[19–23]^ In general, overall postoperative morbidity, mortality and stoma-free survival rates following HP were equivalent or inferior to those following PADS.^[1,20–25]^

According to recommendation 19 of 2016 World Society of Emergency Surgery (WSES) Guidelines, *Hartmann resection is still advised for managing diffuse peritonitis in critically ill patients and in patients with multiple comorbidities. However in clinically stable patients with no co-morbidities primary resection with anastomosis with or without a diverting stoma may be performed (Recommendation 1 B)*.^[26]^

This instruction is shared by many guidelines, although WSES Guidelines offer an additional choice in treating critically ill patients. Indeed, Recommendation 21 stated: *Damage control surgery strategy may be suggested for clinically unstable patients with diverticular peritonitis (severe sepsis/septic shock) (Recommendation 1 B)*.^[26]^

Nevertheless, no general agreement has yet been reached on DCS in perforated ACD.^[7,27]^ According to our analysis, patients treated with DCS showed a 23% to 74% overall morbidity rate, a 0% to 20% 30-day mortality rate and 7% to 33% follow-up overall mortality rate, in addition to a 0% to 33% definitive stoma rate.^[11–18]^ The latter result would suggest a potential advantage of DCS over HP.

However, our findings must be carefully taken into account. Indeed, retrospective quality of most analyzed studies and population heterogeneity become clear in patient selection criteria. Although Hinchey III/IV represented inclusion criteria in all studies, clinical presentation was openly sepsis/septic shock or organ failure in less than a quarter individual populations (see Table 2, Clinical presentation), with the only exception of Tartaglia et al, who just enrolled patients with septic shock/organ failure.^[11–18]^

Differences in sepsis/septic shock terminology need to be taken into account. Two out of 8 studies adopted The Third International Consensus Definitions for Sepsis and Septic Shock (Sepsis-3).^[17,18,28]^ Three studies followed German Sepsis Association S-2k guidelines based on the definitions of American College of Chest Physicians/Society of Critical Care Medicine Consensus Conference.^[13–15,29]^ The remaining 3 studies did not report what definitions they had adopted.^[11,12,16]^

Application of DCS principles is based on clinical assessment of a patient with trauma, who is physiologically decompensated, as it is determined by the phase “lethal triad” in hemorrhagic shock: acidosis, coagulopathy, and hypothermia.^[27,30]^ Patients with decompensated trauma should be treated immediately, in order to avoid progression to irreversible physiological exhaustion and death.^[27,30]^ In this case, abbreviated operations allow stabilization, correction, and re-evaluation of physiological imbalances at ICU.^[27,30]^

Likewise, patients undergoing general emergency surgery might experience a decompensated, almost irreversible, physiological exhaustion, and subsequent death.^[6,10,27,30]^ As in trauma lethal triad represents a combination related to patients with hemorrhagic shock – less frequently to patients undergoing general emergency surgery – reference to it might be considered as inappropriate during decision making process for patients undergoing general emergency surgery.^[6,10,27,30]^ For those patients, clinician's decision is mainly based on septic shock's consequences.^[6,10,27,30]^

A deeper analysis of our results, however, highlights how DCS might have represented overtreatment in good portion of ACD general population.^[31,32]^ Taking into account the small number of patients with sepsis/septic shock/organ failure and the great number of Hinchey III patients, we could assume that many enrolled patients belonged to Hinchey III, being hemodynamically stable and without sepsis/septic shock at clinical presentation.^[31,32]^ Therefore, we deem it possible to assume that Authors have often chosen DCS as an alternative to HP or PADS, rather than considering it as an effective measure to overcome patient's potentially lethal criticality.

In Hinchey III patients, who are hemodynamically stable and without sepsis/septic shock at clinical presentation, laparoscopic lavage might represent a more correct method than DCS, which is a more invasive strategy.^[33–35]^ Some Authors consider laparoscopic lavage as one possible strategy of damage control aimed at representing a bridge to definitive surgery.^[7]^ In accordance with Moore et al, we believe that laparoscopic lavage should not be equated to DCS.^[7]^

Moreover, as defined by Moore et al, DCS role in emergency surgery is not only controversial but it is often misconcepted as “planned relaparotomy”.^[27]^ Reoperations are performed every 48 hours for “washing,” until abdomen is free from ongoing peritonitis.^[27]^ Then abdomen is closed.^[27]^ Such method probably prevents and/or provides early treatment of secondary infections, thus reducing multiple organ failures and deaths.^[27]^ Increased use of resources and higher risk of both gastrointestinal fistulas and delayed hernias represent drawbacks of planned relaparotomy.^[27]^

In the light of excellent preliminary results confirmed by literature, we underline the need to further analyze outcomes of DCS in patients with acute peritonitis from perforated colonic diverticulitis – possibly randomized, controlled, multicenter trials – by assessing both potential benefits and drawbacks. These trials should analyze DCS patients in comparison with patient populations undergoing HP and / or PRA/PADS. However, correct patient selection is required. In particular, there is a need for:

i) patient populations with sepsis/septic shock/organ failure and
ii) the adoption of an international and standardized definition of sepsis (e.g., The Third International Consensus Definitions for Sepsis and Septic Shock – Sepsis-3).

### 4.1 Limitations

Our systematic review introduces some limitations:

i) the literature search was not extended to non-English-written scientific papers;
ii) reported events were mainly small retrospective series;
iii) populations under analysis showed heterogeneity;
iv) many relevant data were not thoroughly described by the Authors, as reported in Tables 2–4;
v) overlapping of analyzed populations cannot be ruled out either by 3 Perathoner group's manuscripts^[11,12,16]^ or by 3 Sohn group's studies;^[13–15]^
vi) sepsis, septic shock, and organ failure definitions differed among studies or were missing;
vii) data on age, MPI, operative time, ICU stay, and hospital stay were reported in median days or mean days. For all these reasons, direct comparison of results turned out difficult.

## 5 Conclusion

DCS represents a well known strategy for trauma surgeons. At present, it is spreading in general emergency surgery. Its application to ACD patients seems to offer good outcomes with a lower percentage of patients with definitive ostomy, if compared to HP. However, correct definition of DCS eligible patients is paramount in avoiding overtreatment. In accordance to 2016 WSES Guidelines, DCS remains an effective surgical strategy in critically ill patients affected by sepsis/septic shock and hemodynamical unstability.

We strongly believe that further studies are required to refine indications, timing, techniques of DCS, and resuscitation approaches to patients in non-traumatic abdominal emergencies.

## Acknowledgments

We thank Dr. Daniela Masi (AUSL-IRCCS di Reggio Emilia) for support in English editing.

## Author contributions

**Conceptualization:** Maurizio Zizzo.

**Data curation:** Maurizio Zizzo, Carolina Castro Ruiz, Magda Zanelli, Francesca Sanguedolce, Stefano Ascani.

**Formal analysis:** Maurizio Zizzo, Carolina Castro Ruiz, Magda Zanelli, Francesca Sanguedolce, Stefano Ascani.

**Investigation:** Maurizio Zizzo.

**Methodology:** Maurizio Zizzo, Maria Chiara Bassi.

**Project administration:** Maurizio Zizzo.

**Resources:** Maurizio Zizzo, Magda Zanelli, Maria Chiara Bassi.

**Supervision:** Maurizio Zizzo, Valerio Annessi.

**Validation:** Maurizio Zizzo.

**Writing – original draft:** Maurizio Zizzo.

**Writing – review & editing:** Maurizio Zizzo.

## Supplementary Material

Supplemental Digital Content
