# Supplementary material for: Damage control surgery for the treatment of perforated acute colonic diverticulitis: A systematic review
Source: Medicine (Baltimore). 2020 Nov 25;99(48):e23323. doi: 10.1097/MD.0000000000023323 (PMC7710165; doi:10.1097/MD.0000000000023323)
Supplement: Supplemental Digital Content [file medi-99-e23323-s001.doc]

**Table - Supplemental Digital Content** Newcastle-Ottawa Quality Assessment Form for Cohort Studies

| **Author/Year** | **Newcastle-Ottawa Scale** | | | | | | | | | | | |  |
| --- | --- | --- | --- | --- | --- | --- | --- | --- | --- | --- | --- | --- | --- |
|  | **Selection** | | | |  | **Comparability** | |  | **Outcome** | | | **Total** | **Quality** |
|  | 1^a^ | 2^b^ | 3^c^ | 4^d^ |  | 5^e^ | 6^f^ |  | 7^g^ | 8^h^ | 9^i^ |  |  |
| Perathoner et al./2010 [10] | ● | ● | ● | ● |  | - | - |  | ● | - | ● | 6 | Poor |
| Kafka-Ritsch et al./2012 [11] | ● | ● | ● | ● |  | ● | ● |  | ● | ● | ● | 9 | Good |
| Sohn et al./2016 [12] | ● | ● | ● | ● |  | ● | ● |  | ● | - | ● | 8 | Good |
| Sohn et al./2018 [13] | ● | ● | ● | ● |  | ● | ● |  | ● | - | ● | 8 | Good |
| Sohn et al./2018 [14] | ● | ● | ● | ● |  | ● | ● |  | ● | - | ● | 8 | Good |
| Gasser et al./2019 [15] | ● | ● | ● | ● |  | ● | ● |  | ● | - | ● | 8 | Good |
| Brillantino et al./2019 [16] | ● | ● | ● | ● |  | ● | ● |  | ● | - | ● | 8 | Good |
| Tartaglia et al./2019 [17] | ● | ● | ● | ● |  | ● | ● |  | ● | - | ● | 8 | Good |

^a^ Representativeness of the exposed cohort

^b^ Selection of the non-exposed cohort

^c^ Ascertainment of exposure

^d^ Demonstration that outcome of interest was not present at start of study

^e^ Comparability of cohorts on the basis of the design or analysis (adjusted for age)

^f^ Comparability of cohorts on the basis of the design or analysis (adjusted for any other factor)

^g^ Assessment of outcome

^h^ Was follow-up long enough for outcomes to occur

^i^ Adequacy of follow-up of cohorts
